# Correction to “Characterisation of Impaired Wound Healing in a Preclinical Model of Induced Diabetes Using Wide‐Field Imaging and Conventional Immunohistochemistry Assays”

**DOI:** 10.1111/iwj.70142

**Published:** 2024-12-10

M. Saidian, J. R. T. Lakey, A. Ponticorvo, et al., “Characterisation of Impaired Wound Healing in a Preclinical Model of Induced Diabetes Using Wide‐Field Imaging and Conventional Immunohistochemistry Assays,” *International Wound Journal* 16, no. 1 (2019): 144–152, https://doi.org/10.1111/iwj.13005.

Concerns were raised by a third party regarding duplicated sections within Figures 2A and 3A and identical images used for Figures 3A and 4A in the article above. The authors admitted to the image compilation error in Figures 2A and 3A. The duplicated images in Figures 3A and 4A were confirmed to be a publisher error. The authors fully cooperated with the investigation and were able to retrieve most of the underlying raw data.

Although the authors could not provide the original images (the locations of the regions of interest [ROIs] for each wound were not recovered) used in the article, when re‐analysing their raw data, they could confirm the same trends as observed before, therefore the experimental results and corresponding conclusions mentioned in the paper remain unaffected.

The corrected images of Figures 2A, 3A and 4A are shown below.

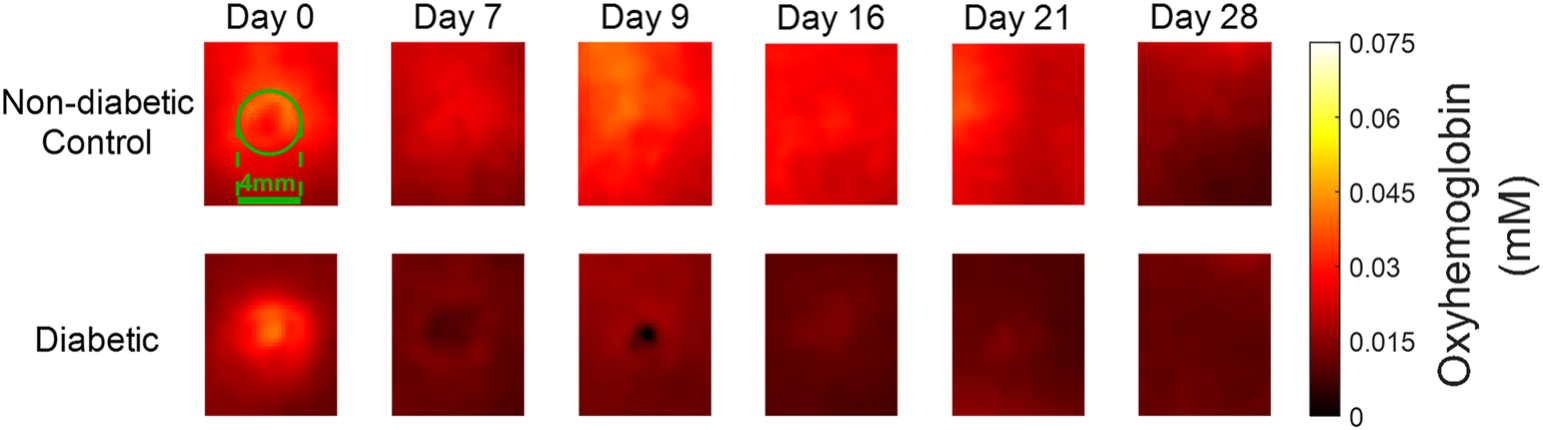

**Figure 2A**

Representative Spatial Frequency Domain Imaging (SFDI) maps of HbO_2_ concentration for one non‐diabetic control rat and one diabetic rat. The yellower the colour, the higher the HbO_2_ concentration, and the darker (redder) colour represents lower HbO_2_ levels.

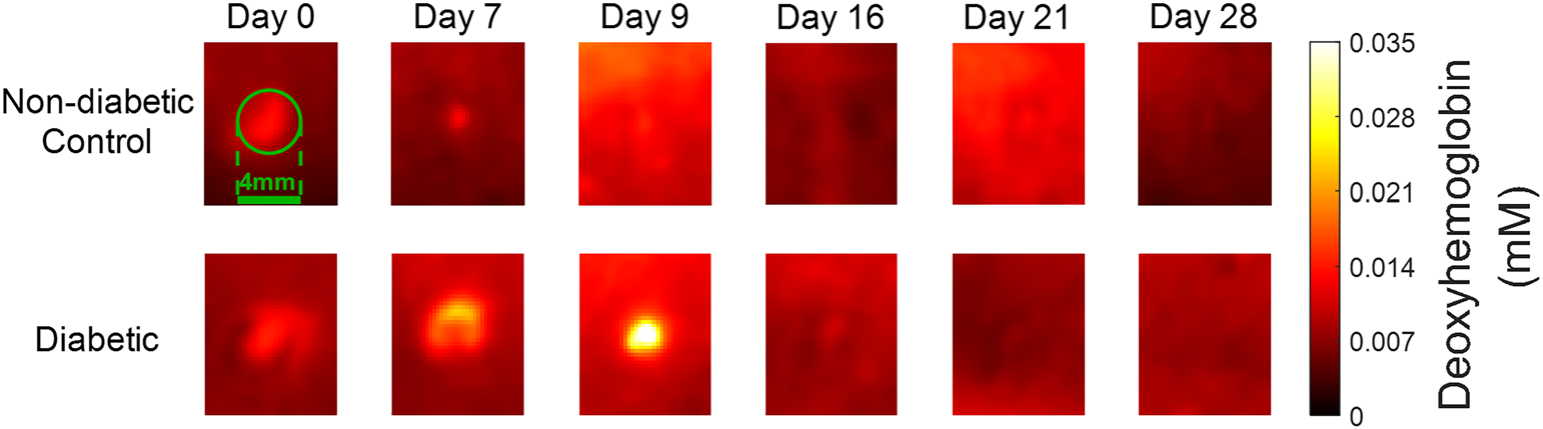

**Figure 3A**

Representative SFDI maps for evaluating the Hb levels for one non‐diabetic control rat and one diabetic rat. The yellower colour indicates higher Hb levels, and the darker (redder) colour represents lower Hb levels.

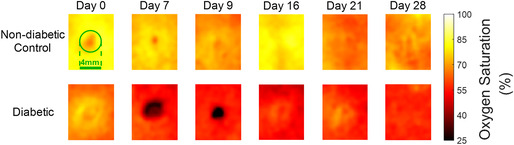

**Figure 4A**

Representative SFDI maps for evaluating oxygen saturation for one non‐diabetic control rat and one diabetic rat. The yellower colour indicates higher oxygen saturation levels, and the darker (redder) colour represents lower oxygen saturation.

The authors also wished to make the following correction to the text, in Section 1. Key Messages inset box:

- Nine CD hairless rats were used, three non‐diabetic control and six diabetic controls, to follow changes in the haemodynamics of wound healing using SFDI.

The authors and the publisher sincerely apologise for this oversight.

